# Influence of Layer Thickness and Raster Angle on the Mechanical Properties of 3D-Printed PEEK and a Comparative Mechanical Study between PEEK and ABS

**DOI:** 10.3390/ma8095271

**Published:** 2015-09-01

**Authors:** Wenzheng Wu, Peng Geng, Guiwei Li, Di Zhao, Haibo Zhang, Ji Zhao

**Affiliations:** 1 School of Mechanical Science and Engineering, Jilin University, Renmin Street 5988, Changchun 130025, China; E-Mails: wzwu@jlu.edu.cn (W.W.); gengpeng13@mails.jlu.edu.cn (P.G.); ligw1411@mails.jlu.edu.cn (G.L.); zhaodi@mails.jlu.edu.cn (D.Z.); 2 Alan G. MacDiarmid Institute, College of Chemistry, Jilin University, Qianjin Street 2699, Changchun 130012, China; E-Mail: zhanghaib@jlu.edu.cn

**Keywords:** 3D printing, polyether-ether-ketone (PEEK), raster angle, layer thickness

## Abstract

Fused deposition modeling (FDM) is a rapidly growing 3D printing technology. However, printing materials are restricted to acrylonitrile butadiene styrene (ABS) or poly (lactic acid) (PLA) in most Fused deposition modeling (FDM) equipment. Here, we report on a new high-performance printing material, polyether-ether-ketone (PEEK), which could surmount these shortcomings. This paper is devoted to studying the influence of layer thickness and raster angle on the mechanical properties of 3D-printed PEEK. Samples with three different layer thicknesses (200, 300 and 400 μm) and raster angles (0°, 30° and 45°) were built using a polyether-ether-ketone (PEEK) 3D printing system and their tensile, compressive and bending strengths were tested. The optimal mechanical properties of polyether-ether-ketone (PEEK) samples were found at a layer thickness of 300 μm and a raster angle of 0°. To evaluate the printing performance of polyether-ether-ketone (PEEK) samples, a comparison was made between the mechanical properties of 3D-printed polyether-ether-ketone (PEEK) and acrylonitrile butadiene styrene (ABS) parts. The results suggest that the average tensile strengths of polyether-ether-ketone (PEEK) parts were 108% higher than those for acrylonitrile butadiene styrene (ABS), and compressive strengths were 114% and bending strengths were 115%. However, the modulus of elasticity for both materials was similar. These results indicate that the mechanical properties of 3D-printed polyether-ether-ketone (PEEK) are superior to 3D-printed ABS.

## 1. Introduction

3D printing is a new integrated manufacturing technology that involves a variety of disciplines. 3D printing has shown excellent potential to reduce both the cycle time and cost of product development [1]. With the development of 3D printing, a large number of processes have been developed that allow the use of a variety of materials and methods [2,3]. Amongst these technologies, one of the most commonly used is fused deposition modeling (FDM) [4,5], a layer-by-layer additive manufacturing technique, based on computer-aided design (CAD) and computer-aided manufacturing (CAM) [6]. The advantages of this technology are as follows [7]: easy material change, low maintenance costs, supervision-free operation, compact size and low working temperature [8]. However, the main disadvantage of FDM is the narrow range of available materials [9]. Many commercial 3D printers can print only acrylonitrile butadiene styrene (ABS) or poly (lactic acid) (PLA). Most parts fabricated by commercial 3D printers are used as demonstration parts, not as working parts. This limits the use of FDM in industrial applications [10].

Polyether-ether-ketone (PEEK), a semi-crystalline thermoplastic material, is an engineering plastic developed by Imperial Chemical Industries (ICI) in 1977 that has high temperature resistance, superior mechanical strength and outstanding chemical stability. In addition, PEEK is biocompatible and ideally suited for use in biomedical applications. As a consequence of these advantages, PEEK can be used in a wide variety of fields, such as the aerospace, automotive, electronics and medicine industries.

The mechanical properties of 3D-printed parts are important indices for evaluating printing quality [11,12,13,14]. Regarding this issue, some work has been carried out to determine the effects of production parameters on the mechanical properties of 3D printed parts. Ismail *et al.* [15] studied the influence of raster angle and orientation on the mechanical properties of ABS printed parts. Tensile and three-point bending tests have been studied. Sood *et al.* [16] focused on the influence of orientation, layer thickness, raster angle, part raster width and raster-to-raster gap. Tymrak *et al.* [17,18] described the influence of orientation, layer thickness and raster-to-raster gap on parts that were printed using several commercial 3D printers. The above research has mainly been concerned with ABS materials.

This paper reports on the mechanical properties of PEEK samples built by a custom-built 3D printing system. PEEK samples were fabricated for two experiments. The first experiment was used to investigate the influence of printing parameters on mechanical properties. The second experiment was used to compare the mechanical properties of PEEK and ABS FDM parts.

## 2. Experimental Section

**Figure 1:**
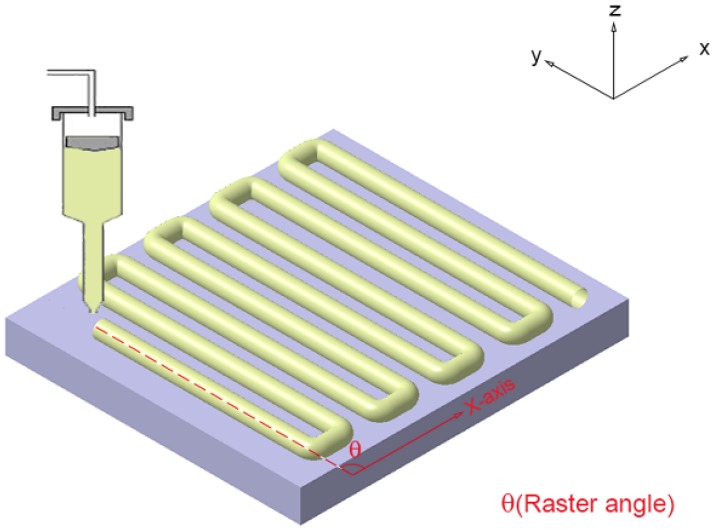
The raster angle of 3D printing.

Mechanical properties of 3D-printed samples can be influenced by many factors, such as layer thickness, raster angle, build orientations, fill pattern, air gap and model build temperature. Layer thickness is the thickness of the layer deposited by the nozzle. Raster angle is the direction of raster with respect to the loading direction of stress, as shown in Figure 1. Air gap is the distance between two adjacent deposited filaments in the same layer. The number of contours is the number of filaments initially deposited along the outer edge. Bead width is the width of the filament deposited by the 3D printer nozzle.

The work described herein investigated the influence of layer thickness (LT) and raster angle (RA) on the mechanical properties of PEEK samples built by a custom-built 3D printing system. Samples with three raster angles (0°, 30° and 45°) and three layer thicknesses (200, 300 and 400 μm) were built by 3D printing of PEEK and tested for tensile, compressive and bending strengths. The printing parameters are shown in Table 1. Build orientation refers to the inclination of part in a build platform with respect to X, Y, Z axis (side, flat and up) [18]. In our study, samples grown up along Z axis and sat down flat during the building process. Fill pattern is the motion path of the 3D printing machine’s liquefier head [19]. During the printing process, the liquefier head move back and forth forming a rectangular structure to fill the entire internal portion of layer. Air gap is the gap between two adjacent rasters on same layer.

**Table 1:** The printing parameters of polyether-ether-ketone (PEEK) 3D printing.

Control Factors			Fixed Factors		
Factor	Level	Unit	Factor	Value	Unit
Layer thickness	200	mm	Build orientation	Y-direction (Flat)	-
	300	mm	Fill pattern	Line	-
	400	mm	Air gap	0	mm
Raster angle	0	°	Number of contours	2	-
	30	°	Nozzle inner diameter	0.4	mm
	45	°			

**Figure 2:**
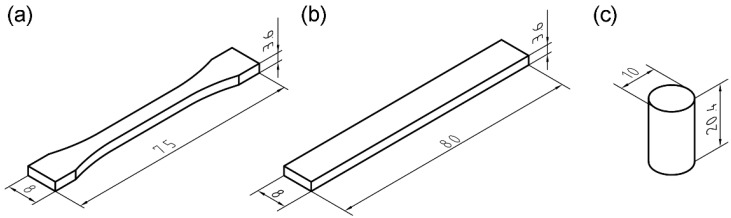
Standard samples for mechanical tests. (**a**) Tensile samples; (**b**) Bending samples; (**c**) Compressive samples.

To investigate the relationships between printing factors and mechanical properties, five samples of each geometry were created, with different layer thicknesses and raster angles, using PEEK material. Identical samples were built using ABS material to compare the mechanical properties of 3D-printed PEEK and ABS parts. The geometric models of the tensile, bending samples and compressive samples were similar to the specifications in GB/T 16421-1996, GB/T 9341-2008 and GB/T1041-2008, respectively. Mechanical test sample models conforming to the relevant mechanical test standards were designed in CATIA V5, and the geometric models were exported as files in stereolithography (STL) format for import by the FDM software. The geometric models are shown in Figure 2.

The experimental samples were made from PEEK and ABS plus^TM^-P430. High-performance PEEK material was obtained from the Jilin University Special Plastic Engineering Research Co. Ltd. (Changchun, China), with a melting point of 334 °C, a glass transition temperature of 143 °C and a melt index of 44 g/10 min. PEEK samples were then built with a custom-built 3D printing system. In this printing machine, a stage platform moves in the X- and Y- axes, and a head including a nozzle tip and nozzle heater moves along the Z-axis, while fusing and depositing material. ABS is inexpensive, has high mechanical strength, and is widely used in the 3D printing industry [20]. ABS plus^TM^-P430 material (Stratasys Inc., Eden Prairie, MN, USA) was used to make ABS samples. ABS is ideal for building 3D parts when combined with uPrint SE 3D Printers (Stratasys Inc., Eden Prairie, MN, USA). ABS samples were built with uPrint SE 3D Printers using default building parameters to obtain optimal mechanical properties. The SR-30 Soluble Support (synthetic thermoplastic polymer) was used as the support material. The mechanical properties of PEEK and ABS materials are shown in Table 2. Testing of mechanical properties of the raw materials were performed on samples manufactured by injection molding.

**Table 2:** Mechanical properties of PEEK and acrylonitrile butadiene styrene (ABS).

Factor	PEEK	ABS
Tensile Strength	100.0 MPa	37.0 MPa
Elastic Limit	72.0 MPa	31.0 MPa
Compressive Strength	118.0 MPa	37.0 MPa
Compressive Modulus	3.8 GPa	2.3 GPa
Bending Strength	163.0 MPa	53.0 MPa
Bending Modulus	4.0 GPa	2.2 GPa

The mechanical tests were carried out on an autograph universal materials testing machine, equipped with a 50-kN load cell. To minimize experimental error, five samples were built and tested under identical conditions for each mechanical test and the mean values were taken as the results. The samples were performed according to the GB/T 16421-1996 Standard and the samples stretched at a strain rate of 1 mm/min. The three-point bending tests were performed according to the GB/T 9341-2008 Standard with the loading rate of 2 mm/min, the span was 64 mm and the head of loading device was composed of steel cylinders 10 mm in diameter. The compressive test was performed according to the GB/T1041-2008 Standard at a loading rate of 2 mm/min.

## 3. Results and Discussion

### 3.1. Influence of Layer Thickness and Raster Angle on Mechanical Properties of PEEK 

Table 3 shows the practical mean values of mechanical tests for the 3D-printed PEEK samples. From Table 3, it can be noted that the samples built with a 300-μm layer thickness had the greatest strengths in all mechanical tests. The strength of samples with a 400-μm layer thickness decreased significantly. We can also see that samples built with raster angles of 0°/90° had the greatest mechanical strengths. Although the layer thickness had a great influence on tensile strength, it had little influence on bending and compressive strengths. Because the compressive sample was cylindrical, there was no effect of the raster angle.

**Table 3:** Mechanical properties in different layer thickness and different raster angles.

Factors		Tensile strength (MPa)	Bending strength (MPa)	Compressive strength (MPa)
Layer Thickness (μm)	200	40.1	52.1	53.6
	300	56.6	56.1	60.9
	400	32.4	48.7	54.1
Raster Angle (°)	0°/90°	56.6	56.1	-
	30°/−60°	41.8	48.5	-
	45°/−45°	43.3	43.2	-

As indicated in Table 3, the tensile strengths of the PEEK samples were significantly affected by the layer thickness and raster angle. In samples printed at raster angles of 0°/90°, the filaments were oriented parallel to the load direction, producing the strongest samples. Similarly, in samples printed in other orientations, there was a finite angle between the printed microstructural elements and the load direction. The filaments were thus subjected to both tensile and shear stresses, leading to failure at low tensile strength.

During compressive tests, the samples undergo pressure aligned in the axial direction and the adjacent layers bear the shear stress because of the additive build up, which can cause the single layers to slide along one another until the sample finally breaks. The compressive strength of printed samples is significantly lower than that of injected materials because the printed samples are subject to flaws, comprising both extensive pores that are initially squeezed out and weak interlayer bonding.

Weak interlayer bonding is responsible for a decrease in bending strength, which can cause welded layers to delaminate. During printing, each new layer will overlay the top of the previous layer before material solidification from the melt occurs, which leads to shrinkage in the previous layer. The residual stresses in the printed samples resulting from the volume shrinkage were examined to determine the weak interlayer bonding. As the layer thickness increases, the accuracy of the overall finished sample geometry decreases and the outline more readily displays the staircase phenomenon.

### 3.2. Comparison of ABS and PEEK Tensile Strengths

**Figure 3:**
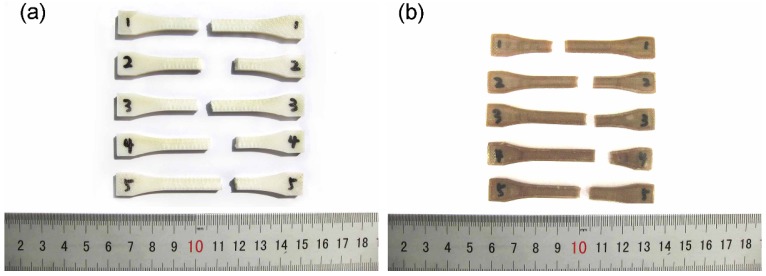
Fractured tensile samples. (**a**) acrylonitrile butadiene styrene (ABS); (**b**) polyether-ether-ketone (PEEK).

**Figure 4:**
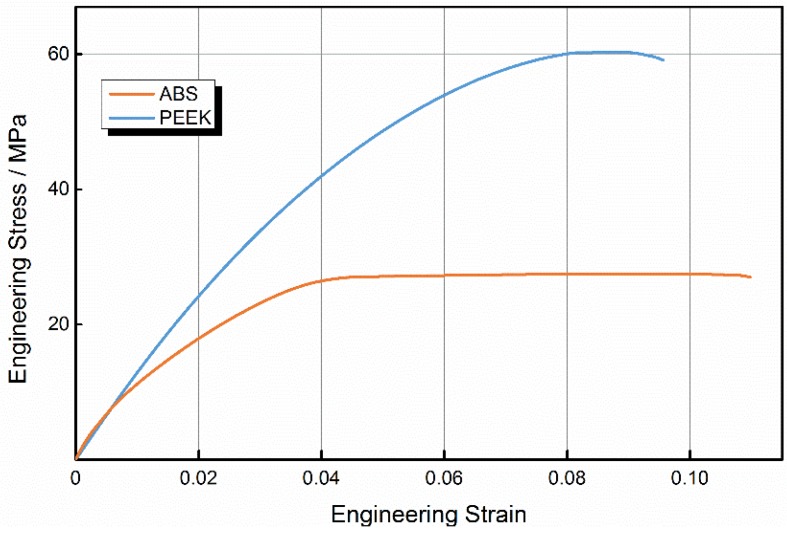
Tensile stress-strain curves of acrylonitrile butadiene styrene (ABS) and polyether-ether-ketone (PEEK).

To evaluate the performance of printed PEEK samples, we built samples from PEEK and ABS using optimal parameters. Figure 3 shows ABS and PEEK samples after the tensile test. Engineering stress-strain curves for PEEK and ABS samples are shown in Figure 4.

The ABS curve includes regions of elastic and plastic deformation, accompanied by necking deformation. The stress-strain behavior under tensile stress was initially nonlinear. After the sample reached peak stress, the resisting stress was almost constant with the increase in strain through shape. It can be concluded that the fracture of ABS occurs mainly via damage to the raster. As the tensile stress increases, the failure will begin at the weakest raster and next weakest raster will break, in sequence, until total failure of the sample. When the stress reaches a certain constant value, a long propagation process occurs in the neck. Craze is the main plastic deformation mechanism of ABS, with a great number of crazes appearing perpendicular to the direction of the tensile loading. Crazes initiate, widen, then suffer breakdown of the raster as tension increases. If crazes extend to both ends of the sample, the sample fails with insignificant necking deformation, because molecular chains initially in an unoriented state transform to a more highly oriented state of necking. The transformation process causes strain hardening, and ensures the uniform expansion of crazes extending to both ends, which is similar to metal deformation hardening caused by uniform deformation.

The PEEK curve clearly differs from the ABS curve. As the load increased, PEEK first yielded at a maximum stress, then necking deformation appeared at the tensile fracture surface and the samples broke after reaching the maximum stress, when the strain reached 87%. In PEEK samples, there was no obvious necking deformation at the tensile fracture surface, and no obvious neck propagation until the fracture of samples after yielding.

**Figure 5:**
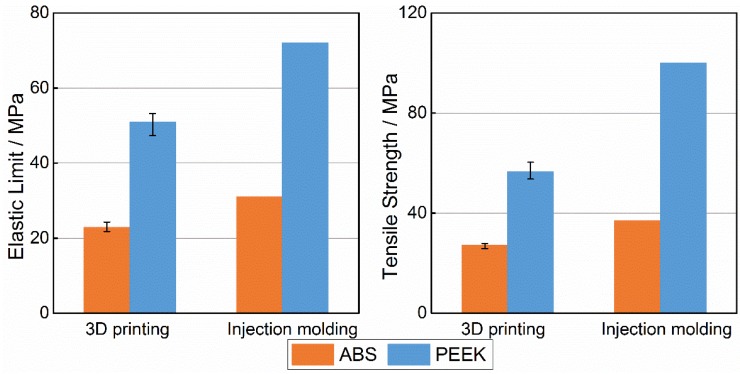
Comparison on tensile property between acrylonitrile butadiene styrene (ABS) and polyether-ether-ketone (PEEK).

Figure 5 shows the average tensile property values for both ABS and PEEK. The elastic limit from five tensile experiments with PEEK was 50.8 MPa; the tensile strength of PEEK was 56.6 MPa, while the elastic limit of ABS was 22.9 MPa, and the tensile strength of ABS was 27.1 MPa. The values of these properties for PEEK were 122% and 108% higher than for ABS. As shown in the figure, the tensile properties of 3D-printed ABS samples were lower than injection-molded ABS by 26.2% for the elastic limit and 26.8% for the tensile strength. Likewise, the tensile properties of 3D-printed PEEK samples were lower than injection-molded PEEK by 29.4% for the elastic limit and 43.4% for the tensile strength. However, the lower strength of 3D-printed samples compared with injection-molded samples can be mainly attributed to the gaps between filaments and the extensive pores within filaments, especially for PEEK samples because of the poor printing quality obtained.

**Figure 6:**
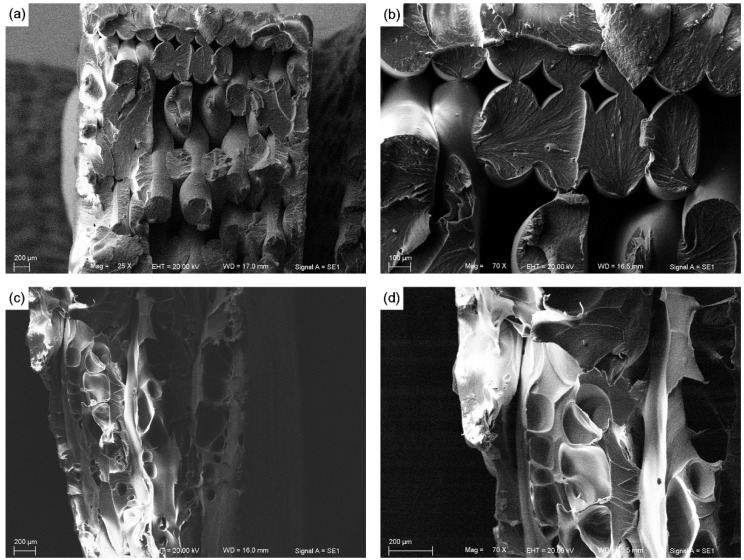
SEM images of fracture cross sections of polyether-ether-ketone (PEEK) and acrylonitrile butadiene styrene (ABS). (**a**) ABS, 25×; (**b**) ABS, 70×; (**c**) PEEK, 25×; (**d**) PEEK, 70×.

Figure 6 shows scanning electron microscopy (SEM) images of fracture cross-sections of PEEK and ABS along the longitudinal direction. These images of the fracture surface show that failure was caused by differing reasons. Although ABS individual rasters had melted together, we can still distinguish every raster in the images, and the fracture of ABS was mainly caused by damage to the rasters pulling and rupturing. As the load force increased, the force per unit area would reach the filaments tensile limit. In the printed samples the fracture would begin approximately at the weakest filament, and the fracture would propagate until the samples failed. The result is that the stress continues to increase and the next weakest raster will fail. By observing the failure surfaces of PEEK samples, we can see that there were no obvious rasters and the samples appeared to have melted into a block. This was most likely caused by a combination of the high extruder temperature and filaments that created significant thermal bonding between both raster and layers, causing greater fusion.

### 3.3. Comparison of ABS and PEEK Compressive Properties

**Figure 7:**
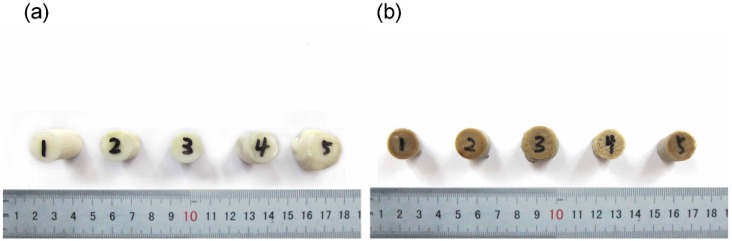
Fractured compressive samples. (**a**) acrylonitrile butadiene styrene (ABS); (**b**) polyether-ether-ketone (PEEK).

**Figure 8:**
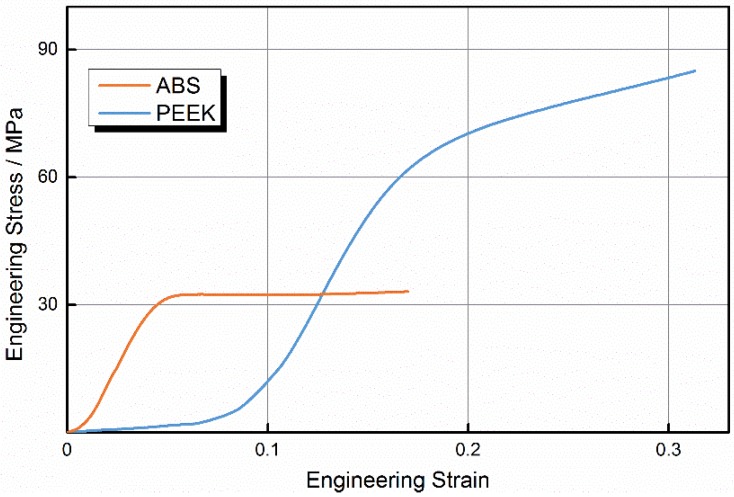
Compressive stress-strain curves of acrylonitrile butadiene styrene (ABS) and polyether-ether-ketone (PEEK).

Figure 7 shows ABS and PEEK samples after compressive tests. Figure 8 shows compressive engineering stress-strain curves for ABS and PEEK. As the figure illustrates, the compressive strength of ABS is obvious, while it is difficult to confirm a unique compressive strength for PEEK. The stress-strain curves for ABS are very similar to the tensile curves for ABS. The stress-stain curve of PEEK is initially linear and the stress increases with the development of significant deformation. With an increase in compressive stress, the curve becomes nonlinear and inelastic. The significant initial deformation appears to result from extensive pores becoming squeezed out. If we could reduce the pores within the printed part, the compressive strength and printing quality of 3D-printed PEEK parts could be substantially improved.

**Figure 9:**
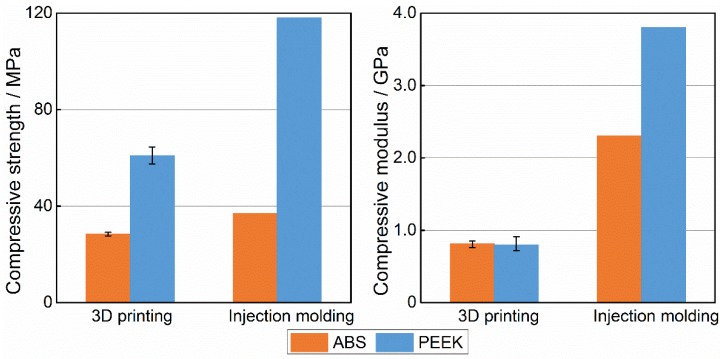
Comparison on compressive property between acrylonitrile butadiene styrene (ABS) and polyether-ether-ketone (PEEK).

The compressive strength and compressive modulus of five experiments are shown in Figure 9. According to Figure 9, the compressive strength of PEEK was 60.9 MPa and the compressive strength of ABS was 28.4 MPa. Thus, the value for PEEK was 114% higher than that for ABS, while the compressive modulus was similar for both. As the figure shows, the 3D-printed samples failed at 76.7% of the value of the injection-molded ABS. Likewise, the injection-molded PEEK failed at 118 MPa, and the 3D-printed samples failed at 76.7% of the injection-molded PEEK. The test results indicate that the compressive strength and modulus of injection-molded ABS samples were higher by 76.9% and 35.3% than those of the 3D-printed ABS samples. Similarly, the 3D-printed PEEK samples failed at 51.6% of the injection-molded PEEK’s compressive strength, and 3D-printed PEEK samples had 79.1% lower compressive modulus than that of injection-molded PEEK.

### 3.4. Comparison of ABS and PEEK Bending Properties

**Figure 10:**
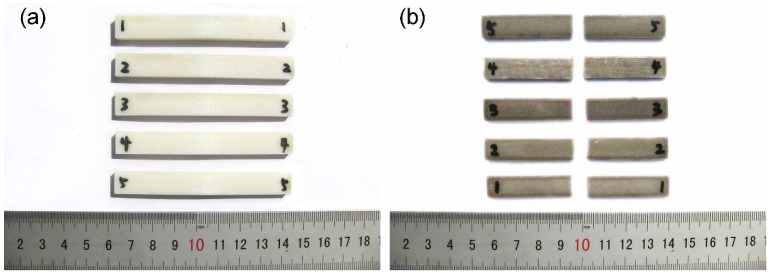
Fractured bending samples. (**a**) acrylonitrile butadiene styrene (ABS); (**b**) polyether-ether-ketone (PEEK).

**Figure 11:**
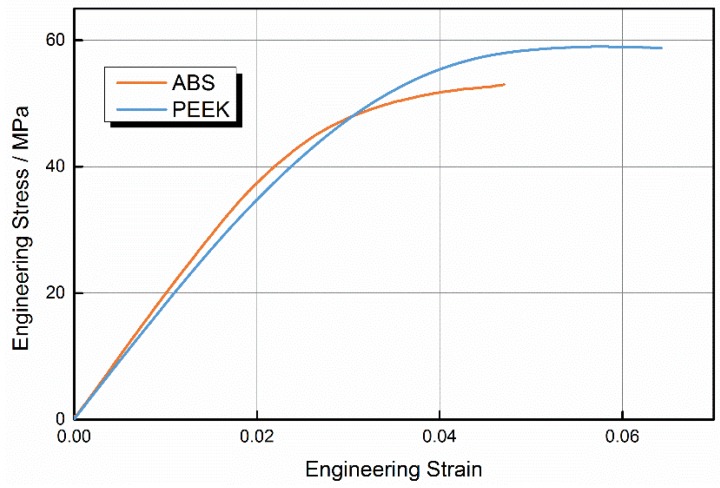
Bending stress-strain curves of acrylonitrile butadiene styrene (ABS) and polyether-ether-ketone (PEEK).

**Figure 12:**
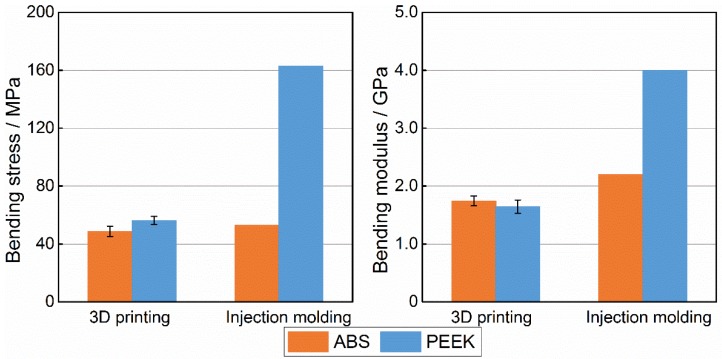
Comparison on bending property between acrylonitrile butadiene styrene (ABS) and polyether-ether-ketone (PEEK).

Figure 10 shows ABS and PEEK samples after three-point bending tests. Figure 11 shows bending engineering stress-strain curves for ABS and PEEK samples. According to the GB/T9341-2008 bending standard, the bending strength is set as the value that causes 3.5% deformation. The bending strength and bending modulus of five experiments are shown in Figure 12. The bending strength of PEEK was 56.2 MPa, 15% higher than that of ABS (48.6 MPa). The bending modulus of PEEK was 1.6 GPa, which was very close to that of ABS. The main reason for the poor flexural property is the weak interlayer bonding. The 3D-printed ABS samples had bending strength and bending modulus reduced by up to 8.2% and 20.8%, respectively, compared with those of injection-molded ABS. The weak interlayer bonding greatly influenced 3D-printed PEEK sample properties, because the bending strength and bending modulus were reduced by up to 65.5% and 58.9%, respectively.

## 4. Conclusions

The aim of this paper was to investigate the effects of raster angle and layer thickness on mechanical properties of 3D-printed samples. The experiments confirmed that raster angle and layer thickness both have a marked effect on tensile, compressive and three-point bending properties. The optimal mechanical properties of PEEK were found in samples with a 300-μm layer thickness and a raster angle of 0°/90°.

Our study also compared the mechanical properties of PEEK and ABS sample parts. Comparing the mechanical properties of ABS and PEEK samples made by 3D printing, it can be concluded that the properties of each part are decreased through 3D-printing compared with those of the raw materials. In this study, the tensile strength of 3D-printed PEEK was about 56 MPa, which is equivalent to that of nylon injection parts. In future studies, the mechanical properties of 3D-printed PEEK parts may be improved by increasing the control accuracy and hardware precision of the 3D-printing system. In this study, the mechanical properties of 3D-printed PEEK samples (tensile, compressive and three-point bending) were higher than those of ABS samples printed by commercial 3D printers. Specifically, the tensile, compression and bending strengths of PEEK samples were higher than those of ABS samples by 108%, 114% and 115%, respectively, while little significant difference was found between the compressive and flexural modulus of PEEK and ABS. Experimental studies and comparative analyses were carried out to study the factors affecting PEEK 3D print forming quality, in the hope of providing reference conditions under which to print PEEK. However, we recognize that there is much work left to do in this area. Further research is needed to reduce pore formation during the printing process and to improve interlayer bonding. PEEK has favorable properties, including excellent mechanical properties in both static and dynamic tests and high chemical resistance [21,22]. It is believed that PEEK may be a significant and promising material for industrial applications of 3D-printed components.
